# Delayed dental development and short stature associated with a novel homozygous *PAPPA2* variant

**DOI:** 10.1210/jcemcr/luag317

**Published:** 2026-10-27

**Authors:** Erdal Kurnaz, Avinaash Vickram Maharaj, Afiya Andrews, Helen L Storr, Sumana Chatterjee, Dinesh Giri

**Affiliations:** Department of Pediatric Endocrinology, Etlik City Hospital, University of Health Sciences, Ankara 06170, Turkey; Centre for Endocrinology, William Harvey Research Institute, Barts and the London School of Medicine, Queen Mary University, London EC1M 6BQ, UK; Centre for Endocrinology, William Harvey Research Institute, Barts and the London School of Medicine, Queen Mary University, London EC1M 6BQ, UK; Centre for Endocrinology, William Harvey Research Institute, Barts and the London School of Medicine, Queen Mary University, London EC1M 6BQ, UK; Department of Paediatric Endocrinology, Bristol Royal Hospital for Children, Bristol BS2 8BJ, UK; Department of Paediatric Endocrinology, Bristol Royal Hospital for Children, Bristol BS2 8BJ, UK; Bristol Medical School, University of Bristol, Queens Road, Bristol BS8 1QU, UK

**Keywords:** short stature, PAPP-A2, elevated IGF-I, delayed dentition, IGF bioavailability

## Abstract

Normal human linear growth depends on intact growth hormone (GH) signaling and insulin-like growth factor-I (IGF-I) bioavailability. In the circulation, IGF-I is largely bound to IGF-binding proteins (IGFBPs), predominantly IGFBP-3 and IGFBP-5, which, together with the acid-labile subunit form a ternary complexes that prolong its half-life. Pregnancy-associated plasma protein-A2 (PAPP-A2) cleaves IGFBP-3 and IGFBP-5, releasing bioactive IGF-I. Pathogenic *PAPPA2* variants impair this process, reducing IGF-I bioavailability despite elevated total IGF-I concentrations. We report a 12.3-year-old Jordanian girl with short stature and delayed dental development. Biochemical evaluation demonstrated markedly elevated serum IGF-I and high-normal IGFBP-3. Comparative genomic hybridization microarray and targeted sequencing of genes associated with postnatal growth failure, including *IGF1R*, were unremarkable. A 6-month trial of recombinant human GH produced no measurable growth acceleration; however, interpretation was limited because treatment was commenced when skeletal maturity was advanced. Extended genetic analysis subsequently identified a novel homozygous *PAPPA2* frameshift variant, c.1223delT (p.L408Rfs*49). This case expands the molecular spectrum of PAPP-A2 deficiency and highlights the importance of *PAPPA2* testing in children with short stature, persistently elevated total IGF-I, and negative *IGF1R* genetic analysis. Delayed dental development may provide an additional clinical clue and help distinguish PAPP-A2 deficiency from other causes of short stature.

## Introduction

Normal linear growth requires an intact and coordinated growth hormone (GH)-insulin-like growth factor-I (IGF-I) axis. Growth hormone promotes growth both directly at the epiphyseal growth plate and indirectly through the stimulation of IGF-I production. In the circulation, IGF-I is largely bound to IGF-binding proteins (IGFBPs), predominantly IGFBP-3 and IGFBP-5. These proteins form a binary complexes with IGF-I and subsequently interact with the acid-labile subunit to form a ternary complexes. These complexes prolong the half-life of IGF-I and regulates its tissue availability [1, 2].

Pregnancy-associated plasma protein-A2 (PAPP-A2; pappalysin 2) is a metalloproteinase that cleaves IGFBP-3 and IGFBP-5, thereby releasing IGF-I from the ternary complexes into its biologically active free form [3, 4]. Consequently, pathogenic variants in *PAPPA2* impair IGF-I bioavailability and disrupt normal growth despite normal or elevated total circulating IGF-I levels.

Several genetic disorders affecting the GH-IGF-I axis are known to cause short stature. These include pathogenic variants in the GH receptor (*GHR*) gene, which cause Laron syndrome [5], and variants in *IGF1*, *IGF1R*, and *IGFALS*, which lead to varying degrees of prenatal and postnatal growth failure [6, 7]. More recently, advances in next-generation sequencing have uncovered additional monogenic causes of short stature involving this pathway [8]. In 2016, Dauber et al [9] identified pathogenic variants in *PAPPA2* as a novel cause of growth failure due to reduced IGF-I bioavailability. Since then, PAPP-A2 deficiency has emerged as a rare but important diagnostic consideration in children previously considered to have idiopathic short stature.

Only a limited number of patients with *PAPPA2* variants have been reported to date. We describe a girl with short stature, elevated total IGF-I and IGFBP-3 concentrations, and delayed dental development, in whom genetic testing identified a novel homozygous frameshift variant in *PAPPA2*.

## Case presentation

A 12.3-year-old girl of Jordanian origin was referred to the pediatric endocrine clinic for evaluation of short stature. She had also been under dental follow-up for approximately 3 years because of generalized delayed dental development. She was born at term with a birth weight of 2.5 kg (−2.3 standard deviation score [SDS]) to nonconsanguineous parents. Her birth weight was therefore consistent with being small for gestational age (SGA) based on birth weight. Birth length and head circumference were not available, so prenatal linear growth could not be assessed. There was no significant past medical history, and she had no symptoms suggestive of chronic systemic disease.

At presentation, her height was 134.3 cm (−2.59 SDS), weight was 36.6 kg, and body mass index was 20.1 kg/m^2^ (+0.59 SDS). Bone age, assessed using the Tanner–Whitehouse 3 (TW3) standards by BoneXpert, was 14.5 years at a chronological age of 13.7 years and was therefore advanced (Fig. 1). Her mid-parental height was 157.2 cm, corresponding to the 9th to 25th centile (−0.9 SDS). She had 2 older sisters, aged 17 and 15 years, both of normal stature. On physical examination, no obvious dysmorphic features were identified, and fifth-digit clinodactyly was not documented clinically; the apparent curvature of the fifth digit on Fig. 1 may be positional. Pubertal development was consistent with Tanner stage IV breast development, and she had not yet attained menarche.

**Figure 1:**
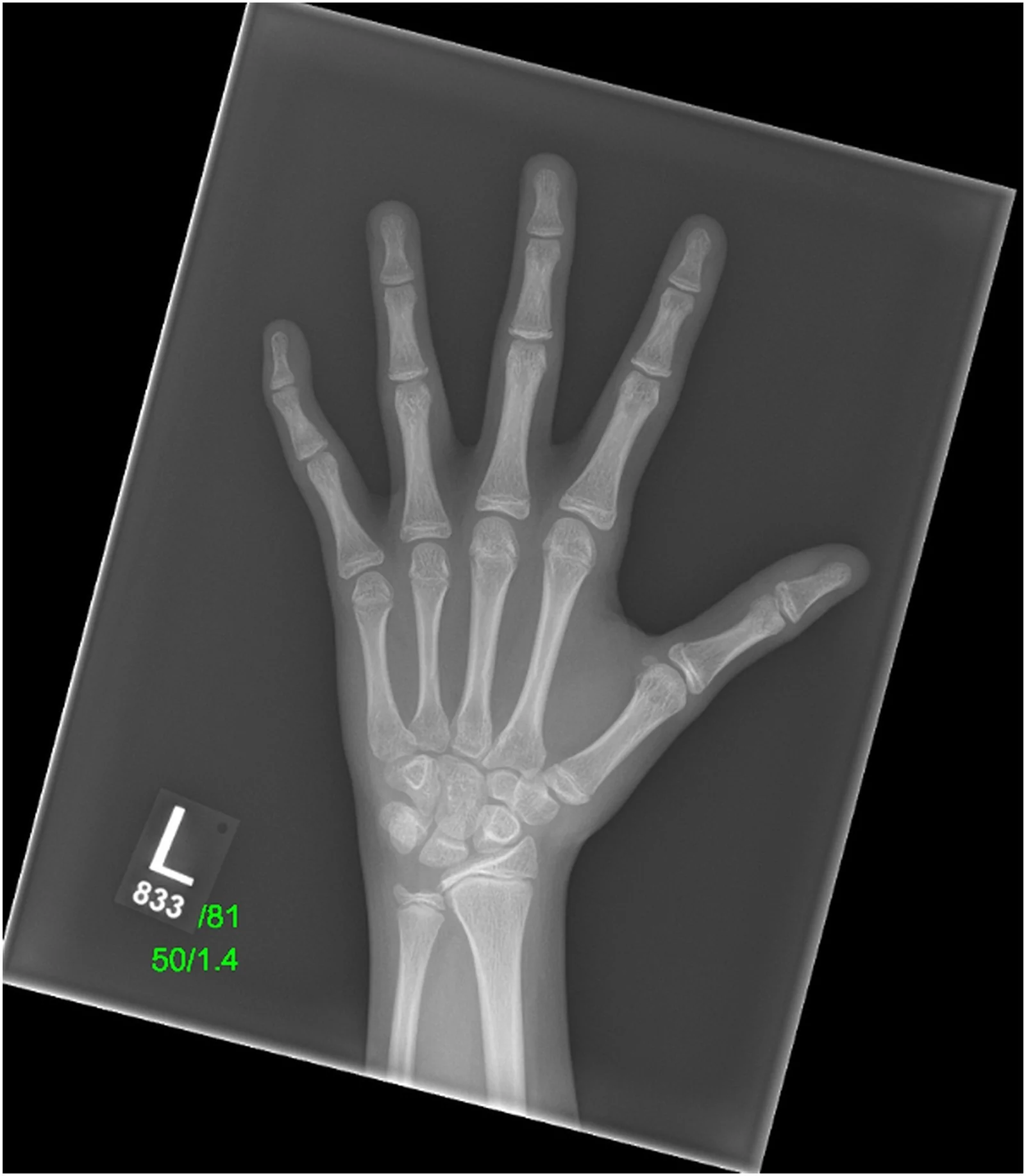
Bone age according to TW3 standards calculated by BoneXpert was 14.5 years at a chronological age of 13.7 years. Fifth-digit curvature may be positional; clinodactyly was not documented clinically.

## Diagnostic assessment

Initial laboratory evaluation revealed normal thyroid function, with a serum thyroid-stimulating hormone concentration of 1.02 µIU/mL (SI [International System of Units]: 1.02 mIU/L) (reference range, 0.27-4.2 µIU/mL [SI: 0.27-4.2 mIU/L]) and free thyroxine concentration of 1.15 ng/dL (SI: 14.8 pmol/L) (reference range, 0.93-1.71 ng/dL [SI: 12-22 pmol/L]). Serum IGF-I was markedly elevated at 1401.29 ng/mL (SI: 183.2 nmol/L) (+4.9 SDS; reference range, 152-691 ng/mL [SI: 19.9-90.3 nmol/L]). Serum IGFBP-3 was high-normal at 7.1 µg/mL (SI: 7.1 mg/L) (+1.9 SDS; reference range, 2.9-8.7 µg/mL [SI: 2.9-8.7 mg/L]).

Comparative genomic hybridization microarray analysis did not identify any pathogenic copy number variants. Targeted sequencing of genes associated with postnatal growth failure, including *IGF1R*, also failed to reveal a causative pathogenic variant.

Subsequent analysis was undertaken by the GRASP (Genetic Research Analysing Short Patients) genetic sequencing research study at Queen Mary University of London using an extended custom short stature gene panel [10], which identified a novel homozygous frameshift variant in *PAPPA2*, c.1223delT (p.L408Rfs*49). Her parents were unaffected obligate heterozygotes. The variant was absent from the Genome Aggregation Database and was classified as pathogenic according to American College of Medical Genetics and Genomics criteria.

## Treatment

Given the biochemical profile and growth pattern, she underwent a trial of recombinant human GH (rhGH). Following 6 months of treatment, there was no clinically meaningful improvement in growth: growth velocity remained suboptimal (Fig. 2), and height SDS declined by 0.21. However, the trial began when bone age was already 14.5 years, indicating limited residual growth potential. Advanced skeletal maturity therefore substantially limits the interpretation of the apparent lack of response, which should not be taken as evidence of intrinsic GH unresponsiveness.

**Figure 2:**
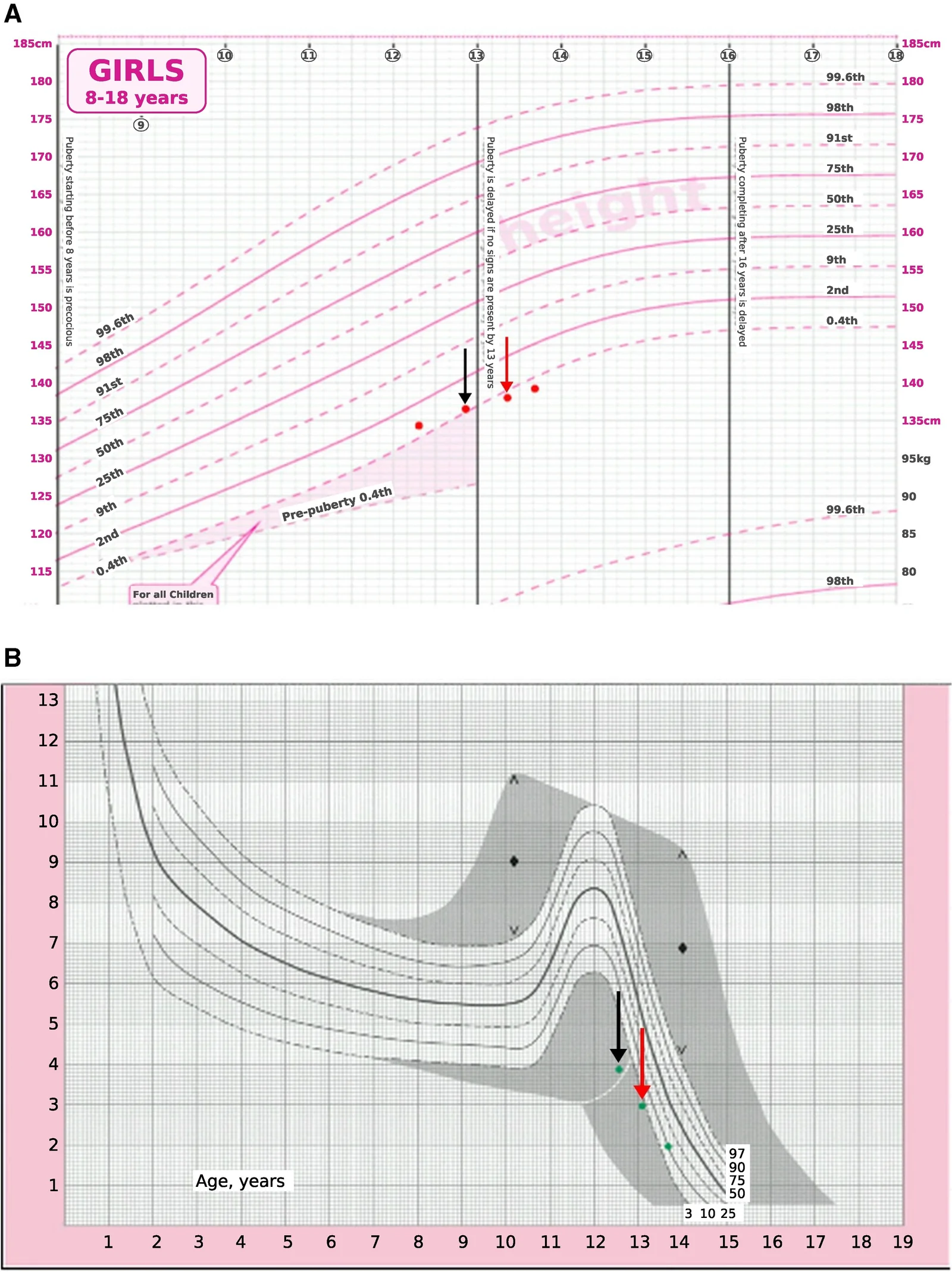
UK-WHO growth charts showing growth during the 6-month rhGH trial. (A) UK-WHO girls aged 8-18 years height chart, with a black arrow showing height before rhGH therapy and a red arrow showing height after 6 months of rhGH therapy. (B) Height velocity chart, with a black arrow showing height velocity before rhGH therapy and a red arrow showing height velocity after 6 months of rhGH therapy. Abbreviation: WHO, World Health Organization.

## Outcome and follow-up

Thereafter, the patient attained menarche at the age of 13 years, and her growth velocity declined to less than 2 cm/year, indicating near-completion of linear growth. Her near-adult height was 140.1 cm. Given her postmenarchal status and proximity to adult height at the time of molecular diagnosis, treatment with recombinant human IGF-1 (rhIGF-I) was not initiated.

## Discussion

Pregnancy-associated plasma protein-A2 deficiency is a rare cause of growth failure, with only a small number of cases reported in the literature [9, 11, 12]. The first description by Dauber et al [9] in 2016 reported 2 affected siblings from a Spanish family and 3 affected siblings from a Palestinian family, with varying degrees of postnatal growth retardation (height SDS, −3.8 to −0.96). Subsequently, in 2021, Babiker et al [11] described 2 affected brothers from Saudi Arabia with height SDS values of −3.6 and −3.3, respectively. Also in 2021, Bertoli-Avella et al [12] identified 2 patients with novel homozygous nonsense variants in *PAPPA2* through large-scale exome/genome sequencing.

The reported phenotype of PAPP-A2 deficiency is relatively consistent. Affected individuals are shorter than expected for target height, with progressive growth failure characterized by declining height velocity over time. Some patients also demonstrate subtle dysmorphic features, including a small chin, microcephaly, long, thin digits, and delayed dental eruption [9, 11]. Our patient shared short stature well below the familial target range and delayed dental development. Her term birthweight was −2.3 SDS and therefore SGA by birthweight; birth length, head circumference, and longitudinal early-infancy growth data were unavailable, so prenatal linear and early postnatal growth could not be characterized. Notably, her delayed dental development was unexpected in relation to Tanner stage IV pubertal development and an advanced hand bone age. Dental eruption abnormalities have not, to our knowledge, been specifically characterized in *Pappa2*-null mice; however, *Pappa2* deletion produces disproportionately smaller and altered mandibles, and reduced local IGF-I bioavailability provides a biologically plausible link with dentoalveolar development [3, 13].

Pubertal progression in PAPP-A2 deficiency appears variable. Some patients enter puberty at expected ages, whereas others may have delayed pubertal onset [9, 11]. Even when puberty is spontaneous and appropriately timed, the pubertal growth spurt may be suboptimal, resulting in reduced near-adult height. This was evident in our patient, who progressed through puberty but failed to show a significant pubertal increase in height velocity. The rhGH trial cannot clarify treatment responsiveness because it was undertaken when skeletal maturity was advanced and residual growth potential was limited.

The biochemical phenotype of PAPP-A2 deficiency is distinctive and clinically informative. Affected patients typically have elevated total serum IGF-I and IGFBP-3 levels but reduced free IGF-I. As a result, tissue exposure to active IGF-I is reduced despite elevated total circulating levels. This biochemical pattern is especially important in the differential diagnosis of short stature with elevated IGF-I, an uncommon clinical scenario.

One important differential diagnosis is dysfunction of the IGF-I receptor, encoded by *IGF1R*. Heterozygous variants in *IGF1R* can cause partial resistance to IGF-I and are associated with intrauterine growth restriction and postnatal growth failure, often accompanied by elevated serum IGF-I concentrations [7]. Homozygous *IGF1R* variants have also been reported in patients with severe pre- and postnatal growth failure and high IGF-I levels [14]. In our patient, however, *IGF1R* genetic analysis was negative, and identification of a *PAPPA2* variant provided a biologically coherent explanation for the discordance between elevated total IGF-I and impaired growth.

There are limited data regarding treatment of PAPP-A2 deficiency with rhIGF-I. In one report, rhIGF-I therapy increased growth velocity in 2 affected siblings [15]. In another study, 1 patient discontinued treatment because of pseudotumor cerebri, while another showed an increase in growth velocity from 3.0 to 6.2 cm/year [16]. Longer-term follow-up of 1 of these patients over 5 years showed modest improvement in height SDS, from −2.9 to −2.3, but without true catch-up growth [17]. Taken together, these findings suggest that rhIGF-I may provide some benefit, particularly when initiated earlier in childhood, although the magnitude of response appears limited. In our patient, rhIGF-I therapy was not considered appropriate because she was postmenarchal when the molecular genetic diagnosis was made, with an advanced bone age of 14.5 years and likely near-adult height attainment. Residual growth potential was therefore considered limited, making a clinically meaningful height response unlikely.

This case broadens the molecular spectrum of *PAPPA2*-related growth failure by identifying a novel homozygous frameshift variant, c.1223delT (p.L408Rfs*49). It also reinforces the clinical relevance of delayed dentition as a recurring feature of the condition. Recognition of this phenotype, together with the characteristic biochemical profile of elevated total IGF-I and IGFBP-3, should prompt consideration of *PAPPA2* analysis in children with unexplained short stature, particularly after negative *IGF1R* testing.

In summary, PAPP-A2 deficiency is a rare but important cause of growth failure. Although phenotypic severity may vary, the biochemical pattern is highly suggestive, and delayed dental development appears to be a useful clinical clue. *PAPPA2* testing should be considered in the diagnostic workup of children with short stature and persistently elevated IGF-I concentrations, especially when *IGF1R* analysis is unremarkable.

This case expands the known molecular spectrum of PAPP-A2 deficiency and emphasizes the importance of recognizing this rare diagnosis in children with short stature and paradoxically elevated serum IGF-I levels. Early recognition may facilitate more timely diagnosis and consideration of targeted therapy, such as recombinant IGF-I, before completion of growth.

## Learning points

- Pregnancy-associated plasma protein-A2 deficiency is a rare genetic cause of short stature characterized by reduced IGF-I bioavailability despite elevated total circulating IGF-I levels.
- Affected patients may show progressive growth failure, subtle dysmorphic features, and delayed dental eruption.
- Recombinant human IGF-I therapy may improve growth velocity modestly, although catch-up growth is limited.
- The findings support candidate genetic *PAPPA2* testing in children with short stature and persistently elevated IGF-I levels but a normal *IGF1R*.

## Contributors

All authors made substantial individual contributions to the work. S.C. and D.G. were directly involved in the patient's clinical assessment, diagnostic evaluation, management, and longitudinal follow-up, including communication with the patient and her family, and contributed to the acquisition and interpretation of the clinical data. A.V.M., H.L.S., and A.A. undertook the subsequent genetic analysis through the GRASP genetic sequencing research study at Queen Mary University of London, using an extended custom short stature gene panel. They contributed to the analysis and interpretation of the sequencing results, evaluation of the novel *PAPPA2* variant, genotype–phenotype correlation, and consideration of its diagnostic and clinical implications. E.K. contributed to the conception of the case report and the analysis and interpretation of the clinical findings in the context of the existing evidence. All authors critically reviewed the work for important intellectual content and approved the final version for publication.

## Data Availability

Data sharing is not applicable to this article, as no datasets were generated or analyzed in the current study.
